# Stepwise correlation of *TP53* mutations from pancreaticobiliary maljunction to gallbladder carcinoma: a retrospective study

**DOI:** 10.1186/s12885-021-09000-2

**Published:** 2021-11-19

**Authors:** Satoshi Kawakami, Shinichi Takano, Mitsuharu Fukasawa, Hiroko Shindo, Ei Takahashi, Yoshimitsu Fukasawa, Hiroshi Hayakawa, Natsuhiko Kuratomi, Makoto Kadokura, Naohiro Hosomura, Hidetake Amemiya, Hiromichi Kawaida, Hiroshi Kono, Shinya Maekawa, Daisuke Ichikawa, Nobuyuki Enomoto

**Affiliations:** 1 First Department of Internal Medicine, Faculty of Medicine, University of Yamanashi, 1110 Shimokato, Chuo, Yamanashi, 409-3898 Japan; 2 First Department of Surgery, Faculty of Medicine, University of Yamanashi, Chuo, Yamanashi, Japan

**Keywords:** Gallbladder cancer, Pancreaticobiliary maljunction, *TP53*, Next-generation sequencing

## Abstract

**Background:**

The genetic changes underlying carcinogenesis in patients with risk factors of gallbladder carcinoma (GBC) remains controversial, especially in patients with pancreaticobiliary maljunction (PBM). This study aimed to clarify the association between risk factors of GBC and genetic changes using next-generation sequencing (NGS).

**Methods:**

We retrospectively analyzed resected tissues of 64 patients who were diagnosed with GBC (*n* = 26), PBM [with GBC (*n* = 8), without GBC (*n* = 20)], and chronic cholecystitis, used as a control group (*n* = 10). DNA was extracted from tumors and their surrounding tissues, which were precisely separated by laser-capture microdissection. Gene alterations of 50 cancer-related genes were detected by NGS and compared with clinical information, including PBM status.

**Results:**

The most frequent gene alterations in GBC tissues occurred in *TP53* (50%), followed by *EGFR* (20.6%), *RB1* (17.6%), and *ERBB2* (17.6%). Gene alterations that were targetable by molecular targeted drugs were detected in 20 cases (58.8%). Statistical analysis of gene alterations and risk factors revealed that *TP53* alteration rate was higher in GBC patients with PBM than those without PBM (*p* = 0.038), and the TP53 mutation rates in the epithelium of control patients, epithelium of PBM patients without GBC, peritumoral mucosa of GBC patients with PBM, and tumor tissue of GBC patients with PBM were 10, 10, 38, and 75%, respectively (*p* <  0.01).

**Conclusions:**

*TP53* alteration more than *KRAS* mutation was revealed to underlie carcinogenesis in patients with PBM.

**Supplementary Information:**

The online version contains supplementary material available at 10.1186/s12885-021-09000-2.

## Background

Gallbladder cancer (GBC), which arises from the epithelium of the gallbladder, shows differences in geographic distribution, with an incidence of 1.5 and 27.3 per 100,000 individuals in North and South America, respectively [1, 2]. In Japan, the incident rate and overall 5-year survival rate of GBC are 7 per 100,000 individuals and 39.8%, respectively [3]. To date, early diagnosis and surgery have been the exclusive curative therapy for GBC [3, 4]. Risk factors for GBC include gallstones [5], diabetes mellitus [6], obesity [7], bacterial infection [8–10], smoking [11], alcohol consumption [12], and pancreaticobiliary maljunction (PBM) [13]. PBM is a congenital anomaly defined as a junction of the pancreatic and biliary ducts located outside the duodenal wall, which usually forms a long common channel. Of 2561 registered patients with PBM, GBC was observed in 13.4 and 37.4% of PBM patients with and without biliary dilation, respectively [13]. The high incidence of GBC in patients with PBM merited elucidation of the disease pathology.

The mechanisms of carcinogenesis in PBM are related to the persistent release of pancreatic juice into the bile duct, which induces inflammation in the biliary tract epithelium due to reflux of proteolytic pancreatic enzymes and phospholipase A2, along with mutagenic substances. Exposure to harmful substances induces hyperplastic change in the epithelium of the gallbladder, which leads to dysplasia and subsequently carcinoma [14]. A high frequency of mutations in the genes *KRAS* and *TP53* was reported in GBC tissues of patients with PBM and in the surrounding epithelium of patients with hyperplasia [15, 16]; therefore, hyperplasia of the gallbladder in patients with PBM is considered to be a genetically precancerous state and represents an early event in the multistep carcinogenesis of GBC. However, recent high-throughput sequencing studies have revealed mutations in *KRAS* at a low frequency [17, 18]. In addition, these high-throughput sequencing studies lacked clinical information regarding PBM. Therefore, the genetic background of carcinogenesis of GBC in patients with PBM remains unclear.

In this study, we performed next-generation sequencing (NGS) of 50 cancer-related genes using GBC and normal adjacent tissues separated by laser-capture microdissection (LCM) to elucidate the genetic background of carcinogenesis of GBC in patients with PBM.

## Methods

### Patients and samples

**Fig. 1:**
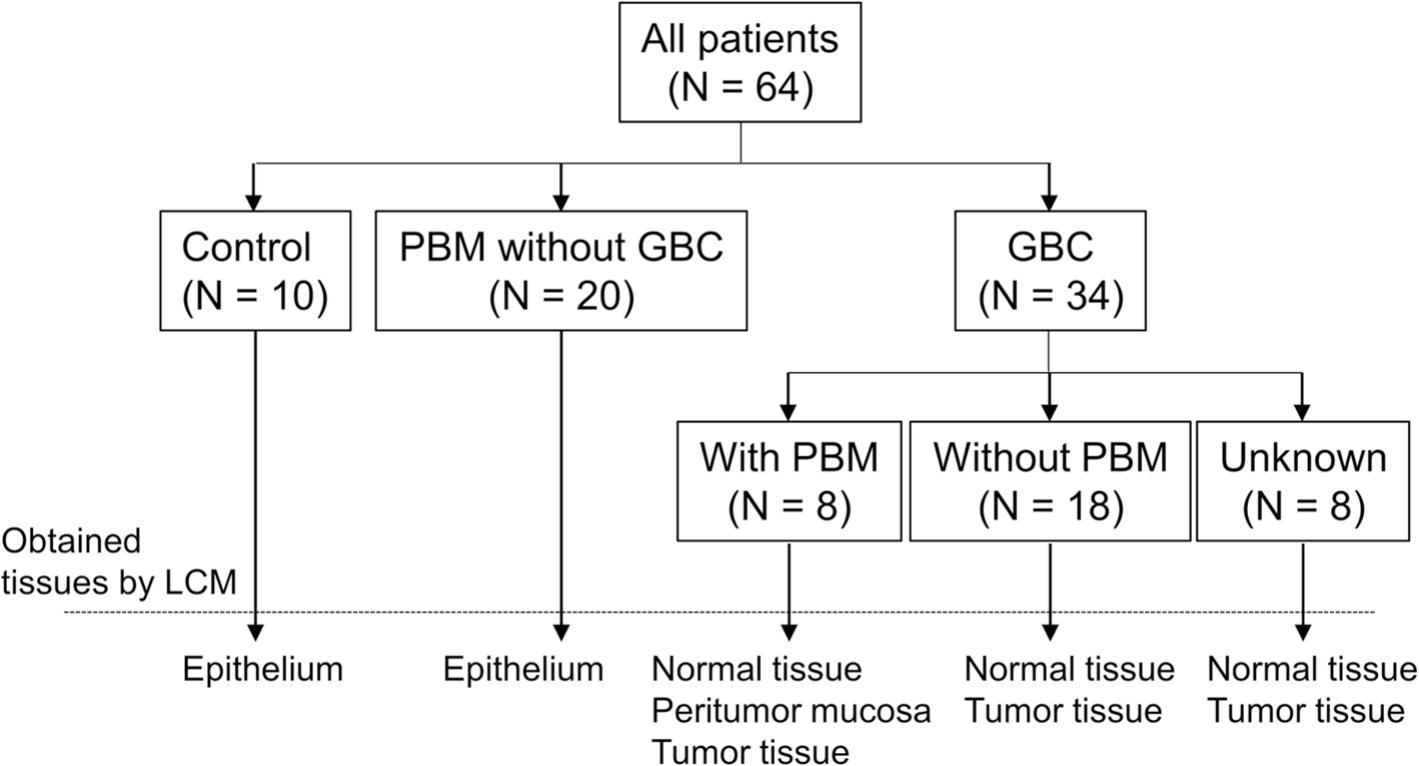
Flow chart of this study

We retrospectively analyzed resected tissues from 64 patients who received surgical resections for gallbladder diseases at Yamanashi University Hospital, and who were diagnosed as GBC-positive (*n* = 26, January 2007–December 2016), PBM-positive [with GBC (*n* = 8), without GBC (*n* = 20), January 2001–December 2017], or diagnosed with chronic cholecystitis, as a control group (*n* = 10, January 2015–December 2016). The flow chart of the study is shown in Fig. 1. PBM was defined as a junction of the pancreatic and bile ducts outside the duodenal wall by magnetic resonance cholangiopancreatography or endoscopic retrograde cholangiopancreatography (ERCP), or amylase levels of the bile obtained during ERCP or surgery no less than 1000 IU/l.

Tissues were obtained from resected specimens in which tumor components and their adjacent normal tissues and/or peritumor mucosa were separated by LCM using an ArcturusXT Laser Capture Microdissection System (Life Technologies, Carlsbad, CA, USA) from 8 μm thick sections of formalin-fixed paraffin-embedded (FFPE) samples. The adjacent normal tissues of the same cases were used as references, such as those from lymph nodes, liver, and gallbladder wall that exist in the muscular layer or deeper, for detecting somatic gene alterations. DNA extraction from the LCM specimens was performed using GeneRead DNA FFPE Kits (QIAGEN, Hilden, Germany) according to the manufacturer’s specifications. The quantities and qualities of the extracted DNA were assessed using a NanoDrop instrument (Thermo Fisher, Waltham, MA, USA) and the Qubit platform (Thermo Fisher). This study was conducted in accordance with the Declaration of Helsinki and was approved by the Human Ethics Review Committee of Yamanashi University Hospital (Receipt numbers: 1326 and 1523). Research data obtained in this study are not shared.

### Genetic analysis by NGS

Genetic analyses of obtained specimens were performed as described previously [19]. Briefly, extracted DNA (10 ng) was amplified using barcode adaptors (Ion Xpress Barcode Adapters 1–96 Kit, Life Technologies) using the Ion AmpliSeq Cancer Hotspot panel version 2 (Thermo Fisher), which contains 207 primer pairs and targets approximately 2800 hotspot mutations located in the following 50 cancer-related genes from the COSMIC database [20]: *ABL1, AKT1, ALK, APC, ATM, BRAF, CDH1, CDKN2A, CSF1R, CTNNB1, EGFR, ERBB2, ERBB4, EZH2, FBXW7, FGFR1, FGFR2, FGFR3, FLT3, GNA11, GNAS, GNAQ, HNF1A, HRAS, IDH1, JAK2, JAK3, IDH2, KDR/VEGFR2, KIT, KRAS, MET, MLH1, MPL, NOTCH1, NPM1, NRAS, PDGFRA, PIK3CA, PTEN, PTPN11, RB1, RET, SMAD4, SMARCB1, SMO, SRC, STK11, TP53*, and *VHL*. The barcoded libraries were amplified using emulsion polymerase chain reaction (PCR) on the Ion Sphere particles. Sequencing was then performed on an Ion Chef System and an Ion Proton Sequencer (Life Technologies) using the Ion PI Hi-Q Chef Kit (Life Technologies) accordingly to manufacturer instructions. Gene mutations and copy number alterations (CNAs) were identified using Ion reporter software version 5.10 (Thermo Fisher). Furthermore, to avoid false-positive variants due to sequencing errors, only mutations and CNAs with mutant allele frequency > 2% (with a sequence read depth of > 100) and copy number ≥ 5 were considered truly present in the tissues.

### Classification of GBC cases according to gene alterations

GBC cases were classified into three groups according to the characteristics of gene alterations. We first defined the cases with more than four gene alterations as the copy number variations (CNV) group, and the other cases were classified as *TP53* mutation and without *TP53* mutation groups using a clustering algorithm in BellCurve for Excel software version 2.20 (Social Survey Research Information Co., Ltd.).

### Actionability assessment of detected variants

The altered genes detected were assessed for their actionability using OncoKB (data version 2.4), which classifies genetic alterations into four levels according to an actionability scale; levels 1–3A indicate standard therapeutic intervention or compelling clinical evidence for the disease, level 3B indicates presence of clinical evidence for another disease, and level 4 indicates presence of compelling biological evidence [21].

### Statistical analysis

All analyses were performed using BellCurve for Excel software version 2.20 (Social Survey Research Information Co., Ltd., Tokyo, Japan). Associations between mutations and clinical variables were evaluated using the Mann–Whitney U test, Fisher’s exact test, chi-square test, and one-way distributed analysis. We used the Cochran–Armitage trend test to determine *TP53* mutation rate in PBM-derived GBC. In all statistical comparisons, a *p*-value of < 0.05 was defined as statistically significant.

## Results

### Patients’ characteristics and quantity of nucleic acids extracted from clinical samples

This study includes patients with chronic cholecystitis (*n* = 10), PBM without GBC (*n* = 20), and patients with GBC (*n* = 34). The average age of each group (mean ± standard deviation [SD]) was 64.5 ± 3.0, 42.4 ± 11.8, and 72.6 ± 9.7 years old, respectively, and the ratio of males to females was 40, 10, and 26.4%, respectively. The rate of accompanying gallstones was 80, 5, and 44%, respectively, and with the rate of smoking was 50, 20, and 15%, respectively. The histological type and Union for International Cancer Control (UICC) stages of GBC were papillary carcinoma (32.4%), well-differentiated adenocarcinoma (35.3%), moderately-differentiated adenocarcinoma (23.5%), and poorly-differentiated adenocarcinoma (8.8%). The UICC stages of GBC patients included 26.4% stage I, 38.2% stage II, 5.9% stage IIIA, 26.4% stage IIIB, 0% stage IVA, and 2.9% stage IVB (Table 1).

**Table 1:** Patient characteristics

Values	(*n* = 34)
Age, mean ± SD, years	72.6 ± 9.72
Sex, *n* (%)	
Male	9 (26.4)
Female	25 (73.5)
Gallstones, *n* (%)	
Yes	15 (44.1)
No	19 (55.9)
Smoking, *n*	
Yes/no/unknown	5/24/5
PBM, *n* (%)	
Yes	8 (23.5)
No	18 (52.9)
Unknown	8 (23.5)
Histological type, *n* (%)	
pap	11 (32.4)
tub1	12 (35.3)
tub2	8 (23.5)
Poor	3 (8.8)
T factor, *n*	
T1/T2/T3/T4	9/19/6/0
Lymph node metastasis, *n*	
Yes/no	10/24
UICC stage	
I/II/IIIA/IIIB/IVA/IVB	9/13/2/9/0/1

*SD* Standard deviation, *PBM* Pancreaticobiliary maljunction, *UICC* Union for International Cancer Control, *pap* papillary adenocarcinoma, *tub1* well-differentiated tubular adenocarcinoma, *tub2* moderately-differentiated tubular adenocarcinoma, *poor* poorly-differentiated adenocarcinoma

Target sequencing of 50 cancer-related genes was performed, and the average (mean ± SD) and median [interquartile range (IQR)] of extracted DNA quantification from tissue samples were 2.3 ± 2.47 ng/sample and 1.68 (0.84–2.88) ng/sample, respectively. The average (mean ± SD) and median (IQR) of the sequence read depths of the samples were 3743 ± 4720 and 2298 (783–4437), respectively.

### Genetic alterations in GBC by NGS analysis

**Fig. 2:**
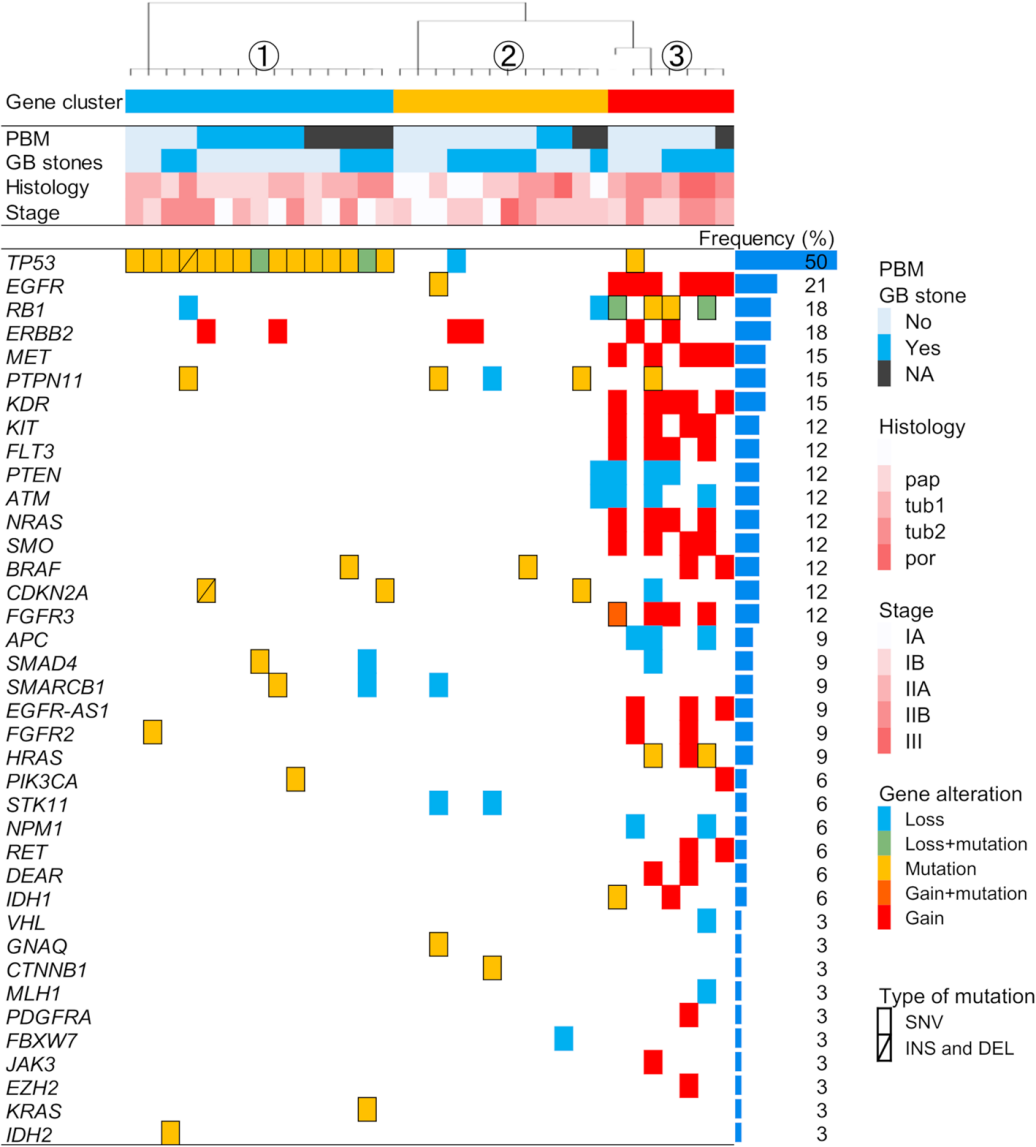
Gene alterations and clinical characteristics of gallbladder carcinoma. The overall view of detected gene alterations in tissues from resected gallbladder cancer specimens is shown. The boxes in the center panel represent detected gene alterations, including mutations and copy number alterations in each case. The left panel shows gene symbols and the frequencies of mutations in each gene; color-coded gene alterations and clinical characteristics are shown in the right panel. The upper panel shows clinical characteristics of each case and a cluster, which was categorized by gene alterations

The most frequent gene alterations in GBC tissues by target sequencing analysis were in *TP53* (50%), followed by *EGFR* (20.6%), *RB1* (17.6%), *ERBB2* (17.6%), *MET* (14.7%), *PTPN11* (14.7%), and *KDR* (14.7%) (Fig. 2). Gene alterations that were targetable by molecular targeting drugs were detected in 20 cases (58.8%), which included alterations in *ATM, BRAF, CDKN2A, EGFR, ERBB2, FGFR2, FGFR3, HRAS, IDH1, IDH2, MET, PIK3CA, PTEN, RET,* and *SMARCB1*. The detected actionable genes are listed in Additional file 1 with categorization using OncoKB.

Next, we classified GBC cases into three groups according to the characteristics of gene alterations. The two criteria for the classification were copy number variations and the clustering algorithm, using which we speculated that mutations in *TP53* were predominantly emphasized. Therefore, we first defined cases with more than four CNVs as the CNV group (group 3 in Fig. 2), and then other cases were classified as *TP53* mutation and without *TP53* mutation groups (groups 1 and 2 in Fig. 2, respectively) using a clustering algorithm in the BellCurve for Excel software version 2.20 (Social Survey Research Information Co., Ltd.). Statistical analysis of three groups revealed that there were differences in neither the presence of gallstones (*TP53* mutant vs. *TP53* normal vs. CNV group: 33.3% vs. 50% vs. 57.1%, *p* = 0.51) nor the history of smoking (*TP53* mutant vs. *TP53* normal vs. CNV group: 10% vs. 16.7% vs. 28.6%, *p* = 0.61), whereas the coexistence rate of PBM was higher in the *TP53* mutation group than in other groups (*TP53* mutant vs. *TP53* normal vs. CNV group: 60% vs. 20% vs. 0%, *p* = 0.027). In addition, the number of altered genes that were targetable by drugs was higher in the CNV group than in other groups [*TP53* mutant vs. *TP53* normal vs. CNV group: 1 (range: 0–1) vs. 0 (range: 0–2) vs. 4 (range: 3–5), *p* <  0.01], as shown in Table 2.

**Table 2:** Comparison of clinical data among clustered groups

	*TP53* mutation group	Without *TP53* mutation group	CNV group	*p*^†^
	(*n* = 15)	(*n* = 12)	(*n* = 7)	
Age, mean ± SD, years	68.3 ± 10.2	74.3 ± 9.2	76.1 ± 10.9	0.100
Sex, male, *n* (%)	4 (26.7)	2 (16.7)	3 (42.9)	0.160
Gallstones, *n* (%)	5 (33.3)	6 (50)	4 (57.1)	0.510
Smoking, *n* (%)^a^	1 (10)	2 (16.7)	2 (28.6)	0.610
PBM, *n* (%)^a^	6 (60)	2 (20)	0	0.027
Histological type				0.130
pap, *n* (%)	6 (40)	5 (41.7)	0	
tub1, *n* (%)	7 (46.7)	4 (33.3)	2 (28.6)	
tub2, *n* (%)	2 (13.3)	2 (16.7)	3 (42.9)	
poor, *n* (%)	0	1 (8.3)	2	
T factor				0.410
T1, *n* (%)	4 (26.7)	3 (25)	0	
T2, *n* (%)	9 (60)	7 (58.3)	4 (57.1)	
T3, *n* (%)	2 (13.3)	2 (16.7)	3 (42.9)	
Lymph node metastasis, *n* (%)	5 (33.3)	2 (16.7)	3 (42.9)	0.440
Number of actionable genes, median (range)	1 (0–1)	0 (0–2)	4 (3–5)	< 0.01

*CNV* Copy number variation, *PBM* Pancreaticobiliary maljunction, *pap* Papillary adenocarcinoma, *tub1* well-differentiated tubular adenocarcinoma, *tub2* Moderately-differentiated tubular adenocarcinoma, *poor* Poorly-differentiated adenocarcinoma

^a^Samples with unknown data were excluded

^†^ *p* values were calculated using chi-square test or one-way distributed analysis

### Accumulation of TP53 mutations in the carcinogenesis of PBM

**Fig. 3:**
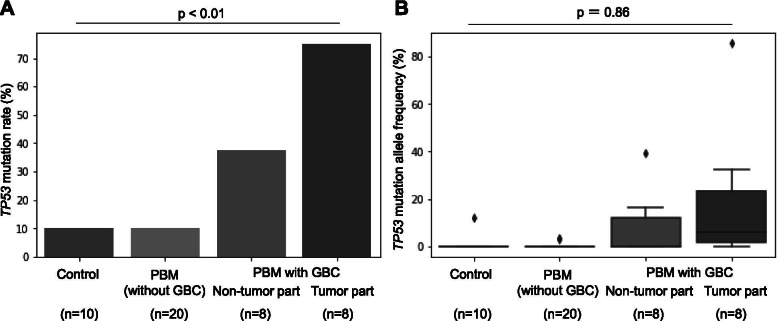
*TP53* mutation in resected tissues. Values of control, PBM (without GBC), non-tumor part of PBM with GBC, and tumor part of PBM with GBC are shown. **A** The percentages of *TP53* mutations in resected tissues are shown. *TP53* mutation rate was high in gallbladder carcinoma (GBC) cases with pancreaticobiliary maljunction (PBM), especially in tumor tissues, as compared to PBM patients without carcinoma or control patients with chronic cholecystitis. **B** The allele frequencies (AFs) of *TP53* mutation are shown as boxplots. AFs of *TP53* increased from control and PBM to PBM with GBC

In response to the association between *TP53* mutation and the presence of PBM, we compared GBC patients with PBM to those without PBM with respect to their clinical and *TP53* mutation status (Table 3). GBC patients with PBM were younger in age than those without PBM (PBM vs. without PBM: 64.6 ± 11.6 years vs. 74.7 ± 9.27 years, *p* = 0.032), and the *TP53* mutation rate was higher in GBC patients with PBM than those without PBM (PBM vs. without PBM: 75% vs. 27.8%, *p* = 0.038). We also analyzed the *TP53* mutation rate and allele frequencies (AFs) of the *TP53* mutation in control and PBM without GBC patients and found that the *TP53* mutation rate was 10, 10, 38, and 75% (Fig. 3A, *p* < 0.01), and the median (mean) AFs of *TP53* mutation were 0 (1.2), 0 (0.3), 0 (8.3), and 6.2 (19.1) (Fig. 3B and Additional file 2, *p* = 0.86) in the epithelium of control patients, epithelium of PBM patients without GBC, peritumoral mucosa of GBC patients with PBM, and tumor tissue of GBC patients with PBM, respectively, which showed a stepwise increase in the *TP53* mutation rate.

**Table 3:** Comparison of GBC with and without PBM

	With PBM	Without PBM	*p*^†^
	(*n* = 8)	(*n* = 18)	
Age, mean ± SD, years	64.6 ± 11.6	74.7 ± 9.27	0.032
Sex, male, *n* (%)	1 (12.5)	7 (38.9)	0.36
Gallstones, *n* (%)	0	10 (55.6)	0.076
Smoking, *n* (%)^a^	1 (20)	2 (11.7)	0.558
Histological type			0.71
pap, *n* (%)	4 (50)	5 (27.8)	
tub1, *n* (%)	2 (25)	7 (38.9)	
tub2, *n* (%)	1 (12.5)	4 (22.2)	
Poor, *n* (%)	1 (12.5)	2 (11.1)	
T factor			0.1
T1, *n* (%)	4 (50)	3 (16.6)	
T2, *n* (%)	4 (50)	10 (55.6)	
T3, *n* (%)	0	5 (27.8)	
Lymph node metastasis, *n* (%)	2 (25)	7 (38.9)	0.667
*TP53* mutations, *n* (%)	6 (75)	5 (27.8)	0.038
Number of actionable genes, *n* (%)	3 (37)	12 (66.7)	0.164

*GBC* Gallbladder carcinoma, *PBM* Pancreaticobiliary maljunction

^a^Samples with unknown data were excluded

^†^ *p* values were calculated using Mann–Whitney U test, Fisher’s exact test, and chi-square test

Based on the result that a PBM with the *TP53* mutation may be a risk for developing GBC, we compared clinical features, such as age, sex, presence of gallstones, smoking, and findings of hyperplasia of the gallbladder mucosa. Results showed that cases of PBM with the *TP53* mutation were older (Table 4).

**Table 4:** Comparison between PBM with and without *TP53* mutations

	*TP53* mutations	Without *TP53* mutations	*p*^†^
	(*n* = 2)	(*n* = 18)	
Age, mean ± SD, years	59.5 ± 4.95	40.4 ± 10.8	0.036
Sex, male, *n* (%)	0 (0)	2 (11.1)	1
Gallstones, *n* (%)	0 (0)	1 (5.6)	1
Smoking, *n* (%)^a^	1 (100)	3 (20)	0.25
Hyperplasia, *n* (%)	0 (0)	13 (72.2)	0.11

*SD* Standard deviation, *PBM* Pancreaticobiliary maljunction

^a^Samples with unknown data were excluded

^†^*p* values were calculated using Mann–Whitney U test, Fisher’s exact test, and chi-square test

## Discussion

In this study, we showed gene alterations in GBC using NGS of 50 cancer-related genes and classified the cases into three groups, which revealed a close association between the *TP53* mutation and PBM. We then analyzed the relationship between the *TP53* mutation rate and PBM by adding control cases and cases of PBM without cancer and revealed a stepwise increase in the *TP53* mutation rate, from control cases to GBC cases with PBM.

The *TP53* mutation rates were high in PBM-derived GBC and its surrounding mucosa, which indicated that mutations in *TP53,* rather than *KRAS*, were associated with carcinogenesis of PBM-derived GBC. PBM is considered a risk factor for GBC through a mechanism wherein harmful substances produced by refluxed pancreatic juice come into contact with the bile duct, which induces hyperplastic change in the epithelium of the gallbladder, leading to dysplasia and finally carcinoma [14]. Genetic changes in *KRAS* and *TP53* have been reported as underlying mechanisms of PBM-derived GBC [15, 16]; however, mutation in *KRAS* was detected in only one case (2.9%) in our study, with an unknown PBM status. Although we cannot ascertain why our study is inconsistent with previous ones, recent studies on high-throughput sequencing of biliary tract cancers might provide clues [17, 18]. The gene alteration rates of *KRAS* in GBC were 0 and 7.8% using whole-exome sequencing in patients in Japan [17] and China [18], respectively, which are lower than previous reports on *KRAS* mutation rates of 38% (15 of 39) [15] and 59% (30 of 51) [22] in patients using low-throughput methods of PCR-SSCP and PCR-RFLP with direct sequencing, respectively. The *KRAS* mutation rate seems to depend on the detection method; that is, low-throughput methods tend to detect high mutation rates compared with recent high-throughput sequencing methods [1], which suggest that low-throughput methods might have higher false-positive rates. In addition, our genetic analysis using target sequencing was reliable as we were able to detect *KRAS* mutations in pancreatic tumor samples at a high frequency as in previous studies [19, 23]. In contrast, the high mutation rates of *TP53* in PBM-derived GBC are consistent with those in previous reports [1], although the issue concerning the timing of alterations in *TP53* remains controversial. Nagai et al. detected no mutations in *TP53* in noncancerous lesions [24], whereas Matsubara et al. reported that mutations in *TP53* were found in 38.5% (10 of 26 cases) of noncancerous biliary lesions with PBM [16], and Kamisawa et al. reported that mutations in *TP53* were found not only in cases with PBM but also in 22.2% (4 of 18 cases) of noncancerous biliary lesions with a relatively long common channel [25], which was also considered a risk factor for GBC. In our study, we performed target sequencing analysis with accurately separated tissues using LCM methods, with detailed clinical information of GBC cases with and without PBM, which revealed that the *TP53* mutation rate of GBC tissues was higher in cases with PBM than in those without PBM (with PBM vs. without PBM: 75% vs. 27.8%, *p* = 0.038). Furthermore, the *TP53* mutation rate of PBM cases without GBC was equivalent to that of controls, and there was a stepwise increase in *TP53* mutations from control and PBM without GBC to peritumoral parts and tumor parts of GBC cases with PBM (control vs. PBM without GBC vs. peritumoral part vs. tumor part of GBC with PBM: 10% vs. 10% vs. 38% vs. 75%, *p* < 0.01), whereas the median (mean) AFs of *TP53* mutations had a tendency to increase from control and PBM without GBC to the peritumoral parts and tumor parts of GBC cases with PBM (control vs. PBM without GBC vs. peritumoral part vs. tumor part of GBC with PBM: 0 [1.2] vs. 0 [0.3] vs. 0 [8.3] vs. 6.2 [19.1], *p* = 0.86). However, these were not statistically significant due to the small sample size. Taken together, these results suggest carcinogenesis of PBM due to gene alterations in *TP53*. To the best of our knowledge, we are the first to report this finding.

There are multiple clinical implications for the findings of our study. First, we detected targets for molecular targeted drugs, especially in the CNV group, with a median of four targets per case. The targets included *ATM, BRAF, CDKN2A, EGFR, ERBB2, FGFR2, FGFR3, HRAS, IDH1, IDH2, MET, PIK3CA, PTEN, RET,* and *SMARCB1*, which could be targeted by clinically approved drugs, as listed in Additional file 1. This information could be helpful in the selection of an adequate therapy for patients with advanced GBC. In addition, we were able to find actionable gene alterations using 50 cancer-related gene panels that could detect common gene alterations. Second, the finding that the *TP53* mutation was associated with carcinogenesis in patients with PBM may lead to risk stratification for GBC. The current guidelines for PBM recommend prophylactic resection of the gallbladder with and without biliary reconstruction in PBM cases with and without biliary duct dilation, respectively [26], which might impose an invasive surgical burden on young patients or an unnecessary surgery for certain patients with comorbidities. However, the risk diagnoses of PBM cases by examining the *TP53* status using bile juice or blood samples could provide young patients with adequate time for surgery and could avoid unnecessary operations, at least in patients who are not good candidates for surgery. Therefore, establishing liquid biopsy technique to examine the *TP53* status using bile juice or blood samples will be our next theme for the next 5 years.

This study had several limitations. First, the study design was retrospective in nature, and only a small number of cases were recruited from a single center, especially in cases of PBM with GBC. Second, clinical information of some cases was insufficient, especially regarding the presence of PBM, as detailed ERCP examinations were not often conducted in past decades. Third, some concerns may be raised about the reliability of the CNVs detected in this study. The quantities of DNA obtained and the quality of NGS did not differ between the CNV and non-CNV group (Additional file 3). Furthermore, we successfully detected CNVs in pancreatic cancer and gastric cancer previously using the same protocol with the same gene panel as the current study, and validation was performed successfully using the fluorescence in situ hybridization. Therefore, we can confirm that the CNVs detected in this study were reliable.

## Conclusion

In conclusion, we discovered a relationship between PBM-derived GBC and mutations in *TP53* by target sequencing analysis and detailed clinical information, in which we complemented inconsistencies and lack of clinical information with high-throughput sequencing analysis.

## Supplementary Information

**Additional file 1.** Altered genes, which could be drug targets.

**Additional file 2.** Each type of mutation, allele frequency, and NGS read for the TP53 genes.

**Additional file 3.** Comparison of sequence quality between the CNV and non-CNV groups.

## Data Availability

The datasets generated and/or analyzed during the current study are not publicly available due to the lack of consent of the participants to list them in public databases. However, the data are available from the corresponding author on reasonable request.

## Abbreviations

GBC: Gallbladder carcinoma
PBM: Pancreaticobiliary maljunction
NGS: Next-generation sequencing
LCM: Laser-capture microdissection
FFPE: Formalin-fixed paraffin-embedded
CNAs: Copy number alterations
UICC: Union for International Cancer Control
SD: Standard deviation
IQR: Interquartile range
CNVs: Copy number variations
