# Supplementary material for: Stepwise correlation of TP53 mutations from pancreaticobiliary maljunction to gallbladder carcinoma: a retrospective study
Source: BMC Cancer. 2021 Nov 19;21:1245. doi: 10.1186/s12885-021-09000-2 (PMC8605550; doi:10.1186/s12885-021-09000-2)
Supplement: Supplementary file 1 — Additional file 1. Altered genes, which could be drug targets. [file 12885_2021_9000_MOESM1_ESM.pdf]

Additional file 1. Altered genes which could be drug targets

Case (#)	Gene	Type of Alterations	Level of OncoKB	Molecular targeted drugs
23	*SMARCB1*	Oncogenic mutations	4	Tazemetostat
	ERBB2	Amplification	2	Trastuzumab, Pertuzumab, Lapatinib, Neratinib, Capecitabine, Tucatinib, Deruxtecan, Emtansine
26	*BRAF*	Oncogenic mutations	3	Cobimetinib
27	*IDH2*	Oncogenic mutations	2	Enasidenib
34	*PTEN*	Oncogenic mutations	4	GSK2636771, AZD8186
	SMARCB1	Deletion	2	Tazemetostat
35	*PIK3CA*	Oncogenic mutations	2	Fulvestrant + Alpelisib
36	*CDKN2A*	Oncogenic mutations	4	Abemaciclib, Ribociclib, Palbociclib
	ERBB2	Amplification	2	Trastuzumab, Pertuzumab, Lapatinib, Neratinib, Capecitabine, Tucatinib, Deruxtecan, Emtansine
37	*ERBB2*	Amplification	2	Trastuzumab, Pertuzumab, Lapatinib, Neratinib, Capecitabine, Tucatinib, Deruxtecan, Emtansine
	EGFR	Amplification	4	Lapatinib
	FGFR2	Fusions	2	Erdafitinib, Pemigatinib

38	*ERBB2*	Amplification	2	Trastuzumab, Pertuzumab, Lapatinib, Neratinib, Capecitabine, Tucatinib, Deruxtecan, Emtansine
40	*CDKN2A*	Oncogenic mutations	4	Abemaciclib, Ribociclib, Palbociclib
41	*ERBB2*	Amplification	2	Trastuzumab, Pertuzumab, Lapatinib, Neratinib, Capecitabine, Tucatinib, Deruxtecan, Emtansine
42	*HRAS*	Oncogenic mutations	3	Tipifarnib
	FGFR3	Fusions	2	Erdafitinib
	MET	Amplification	2, R2	Crizotinib, Osimertinib, Gefitinib, Erlotinib, Cabozantinib
	EGFR	Amplification	4	Lapatinib
43	*ERBB2*	Amplification	2	Trastuzumab, Pertuzumab, Lapatinib, Neratinib, Capecitabine, Tucatinib, Deruxtecan, Emtansine
	FGFR3	Fusions	2	Erdafitinib
	IDH1	Oncogenic mutations	2	Ivosidenib
44	*FGFR3*	Fusions	2	Erdafitinib
	FGFR3	Oncogenic mutations	4	BGJ398, AZD4547, Erdafitinib
	MET	Amplification	2, R2	Crizotinib, Osimertinib, Gefitinib, Erlotinib, Cabozantinib
	EGFR	Amplification	4	Lapatinib
	IDH1	Oncogenic mutations	2	Ivosidenib
45	*FGFR3*	Fusions	2	Erdafitinib

	MET	Amplification	2, R2	Crizotinib, Osimertinib, Gefitinib, Erlotinib, Cabozantinib
	EGFR	Amplification	4	Lapatinib
	HRAS	Oncogenic mutations	3	Tipifarnib
48	*BRAF*	Fusions	3	Trametinib, Cobimetinib
	EGFR	Amplification	4	Lapatinib
	MET	Amplification	2, R2	Crizotinib, Osimertinib, Gefitinib, Erlotinib, Cabozantinib
	RET	Fusions	2	Selpercatinib, Pralsetinib, Cabozantinib, Vandetanib
49	*BRAF*	Fusions	3	Trametinib, Cobimetinib
	EGFR	Amplification	4	Lapatinib
	MET	Amplification	2, R2	Crizotinib, Osimertinib, Gefitinib, Erlotinib, Cabozantinib
	RET	Fusions	2	Selpercatinib, Pralsetinib, Cabozantinib, Vandetanib
	FGFR2	Fusions	2	Erdafitinib, Pemigatinib
51	*ATM*	Oncogenic mutations	2	Olaparib
	CDKN2A	Oncogenic mutations	4	Abemaciclib, Ribociclib, Palbociclib
53	*FGFR2*	Oncogenic mutations	4	Debio1347, AZD4547, BGJ398, Erdafitinib
55	*BRAF*	Oncogenic mutations	3	Cobimetinib

57	*CDKN2A*	Oncogenic mutations	4	Abemaciclib, Ribociclib, Palbociclib
