# Supplementary material for: Stepwise correlation of TP53 mutations from pancreaticobiliary maljunction to gallbladder carcinoma: a retrospective study
Source: BMC Cancer. 2021 Nov 19;21:1245. doi: 10.1186/s12885-021-09000-2 (PMC8605550; doi:10.1186/s12885-021-09000-2)
Supplement: Supplementary file 2 — Additional file 2. Each type of mutation, allele frequency, and NGS read for the TP53 genes. [file 12885_2021_9000_MOESM2_ESM.pdf]

Additional file 2. Each type of mutation, allele frequency, and read of NGS for the *TP53* genes

	Case No.	Coding	Type of mutations	Read depth	AF (%)
Chronic cholecystitis	62	c.388C>A	missense	181	12.2
PBM without GBC	4	c.292C>T	missense	1397	3.4
	8	c.844C>T	missense	645	2
	8	c.901C>T	missense	672	2.7
	8	c.848G>A	missense	662	3.9
GBC with PBM	24	c.469G>A	missense	228	4.4
(Non-tumor part)	24	c.725G>A	missense	318	16.4
	25	c.574C>T	nonsense	1643	39.3
	58	c.824G>A	missense	368	16.6
GBC with PBM	22	c.839G>A	missense	673	2.8
(Tumor part)	23	c.473G>A	missense	588	2
	24	c.818G>A	missense	188	9.6
	25	c.574C>T	nonsense	940	85.5
	35	c.524G>A	missense	1994	32.3

	36	c.646G>T	missense	1991	20.2
GBC without PBM	26	c.701A>G	missense	1938	29
	27	c.839G>A	missense	938	31.5
	32	c.461G>T	missense	1983	8.3
	37	c.512A>G	missense	1959	50
	47	c.626_627delGA	frameshift deletion	1965	35.4
	47	c.818G>A	missense	824	2.6
	51	c.738G>A	missense	1950	85.2
	52	c.832C>T	missense	1670	23.5
	53	c.496T>G	missense	1959	2.3
	54	c.586C>T	nonsense	1961	19.4
	57	c.638G>A	missense	1984	19.2

GBC, Gallbladder carcinoma; PBM, Pancreaticobiliary maljunction; AF, allele frequency
