# Supplementary material for: Stepwise correlation of TP53 mutations from pancreaticobiliary maljunction to gallbladder carcinoma: a retrospective study
Source: BMC Cancer. 2021 Nov 19;21:1245. doi: 10.1186/s12885-021-09000-2 (PMC8605550; doi:10.1186/s12885-021-09000-2)
Supplement: Supplementary file 3 — Additional file 3. Comparison of sequence quality between the CNV and non-CNV groups. [file 12885_2021_9000_MOESM3_ESM.pdf]

Additional file 3. Comparison of sequence quality between the CNV and non-CNV groups

	CNV group (*n* = 7)	Non-CNV group (*n* = 27)	*p*‡
DNA concentration, mean ± SD, ng/uL			
Tumor part*	2.17 ± 0.6	3.58 ± 2.28	0.11
Non-tumor part†	3.37 ± 6.83	1.10 ± 1.45	0.93
Read depth, mean ± SD, ng/sample			
Tumor part	2998 ± 2964	5523 ± 4214	0.07
Non-tumor part	903 ± 1084	2568 ± 4429	0.06
Read depth, median (range)			
Tumor part	2171 (304–9023)	4456 (692–18782)	0.07
Non-tumor part	510 (0.8–3112)	1765 (7.4–23947)	0.06

CNV, Copy number variation; SD, Standard deviation

*One tumor part that was lower than the detection limit was excluded

†Two non-tumor parts that were lower than the detection limit were excluded

‡p values were calculated using the Mann-Whitney U test
